# Predicting Severity of Huntington's Disease With Wearable Sensors

**DOI:** 10.3389/fdgth.2022.874208

**Published:** 2022-04-04

**Authors:** Brittany H. Scheid, Stephen Aradi, Robert M. Pierson, Steven Baldassano, Inbar Tivon, Brian Litt, Pedro Gonzalez-Alegre

**Affiliations:** ^1^Department of Bioengineering, University of Pennsylvania, Philadelphia, PA, United States; ^2^Center for Neuroengineering and Therapeutics, University of Pennsylvania, Philadelphia, PA, United States; ^3^Department of Neurology, University of Pennsylvania, Philadelphia, PA, United States; ^4^Huntington's Disease Center of Excellence, University of Pennsylvania, Philadelphia, PA, United States; ^5^Department of Neurology, University of South Florida, Tampa, FL, United States; ^6^Department of Radiology, Massachusetts General Hospital, Boston, MA, United States; ^7^Spark Therapeutics, Philadelphia, PA, United States

**Keywords:** movement disorders, biosensors, gait, accelerometer, Huntington's disease (HD), machine learning, wearables

## Abstract

The Unified Huntington's Disease Rating Scale (UHDRS) is the primary clinical assessment tool for rating motor function in patients with Huntington's disease (HD). However, the UHDRS and similar rating scales (e.g., UPDRS) are both subjective and limited to in-office assessments that must be administered by a trained and experienced rater. An objective, automated method of quantifying disease severity would facilitate superior patient care and could be used to better track severity over time. We conducted the present study to evaluate the feasibility of using wearable sensors, coupled with machine learning algorithms, to rate motor function in patients with HD. Fourteen participants with symptomatic HD and 14 healthy controls participated in the study. Each participant wore five adhesive biometric sensors applied to the trunk and each limb while completing brief walking, sitting, and standing tasks during a single office visit. A two-stage machine learning method was employed to classify participants by HD status and to predict UHDRS motor subscores. Linear discriminant analysis correctly classified all participants' HD status except for one control subject with abnormal gait (96.4% accuracy, 92.9% sensitivity, and 100% specificity in leave-one-out cross-validation). Two regression models accurately predicted individual UHDRS subscores for gait, and dystonia within a 10% margin of error. Our regression models also predicted a composite UHDRS score–a sum of left and right arm rigidity, total chorea, total dystonia, bradykinesia, gait, and tandem gait subscores–with an average error below 15%. Machine learning classifiers trained on brief in-office datasets discriminated between controls and participants with HD, and could accurately predict selected motor UHDRS subscores. Our results could enable the future use of biosensors for objective HD assessment in the clinic or remotely and could inform future studies for the use of this technology as a potential endpoint in clinical trials.

## Introduction

Huntington's disease (HD) is a progressive autosomal dominant neurodegenerative disease caused by a CAG trinucleotide repeat expansion within the *HTT* gene. Clinically, HD is marked by abnormal involuntary movements, cognitive decline, and behavioral changes. Presently, disease onset is identified by the emergence of characteristic abnormal motor findings during clinical examination. The Unified Huntington's Disease Rating Scale (UHDRS) is the current gold standard used to communicate disease severity and consists of a four part battery of assessments measuring motor function, cognitive function, behavioral abnormalities, and functional capacity (1). The UHDRS Total Motor Score (UHDRS-TMS) is a 124-point scale consisting of multiple subscores rated from zero to four (e.g., maximal chorea-left leg, bradykinesia). Although the UHDRS-TMS assessment has acceptable interrater reliability, it is ultimately subjective and its accuracy depends on the clinical experience of the rater (1–3).

Objective measurements of disease characteristics are important for reliably assessing disease progression, both for clinical management and for evaluating the efficacy of novel candidate therapies. Furthermore, acquiring objective data more frequently and in settings other than the clinic, without the necessity of a clinician, may ultimately provide more sensitive biomarkers to inform clinical decision-making and for use in clinical trials. Numerous tools to this end have been investigated, including wearable devices as well as mobile smartphone applications, in the context of monitoring a range of neurological conditions (4–19).

Prior studies have attempted to characterize motor features in HD using various configurations of wearable sensors by recording longitudinal data (19, 20), recording complex observed tasks (18), or by focusing on a particular body region (16, 17, 21). Our objective was to develop an automated scoring method using data from simple assessments to provide point measurements of motor features in HD. The present study applies machine learning methods to automatically classify participant disease status and to predict seven specific UHDRS-TMS subscores (left and right arm rigidity, total chorea, total dystonia, bradykinesia, gait, and tandem gait) using data obtained from wearable sensors worn during simple, brief tasks.

## Methods

### Data Collection

**Figure 1:**
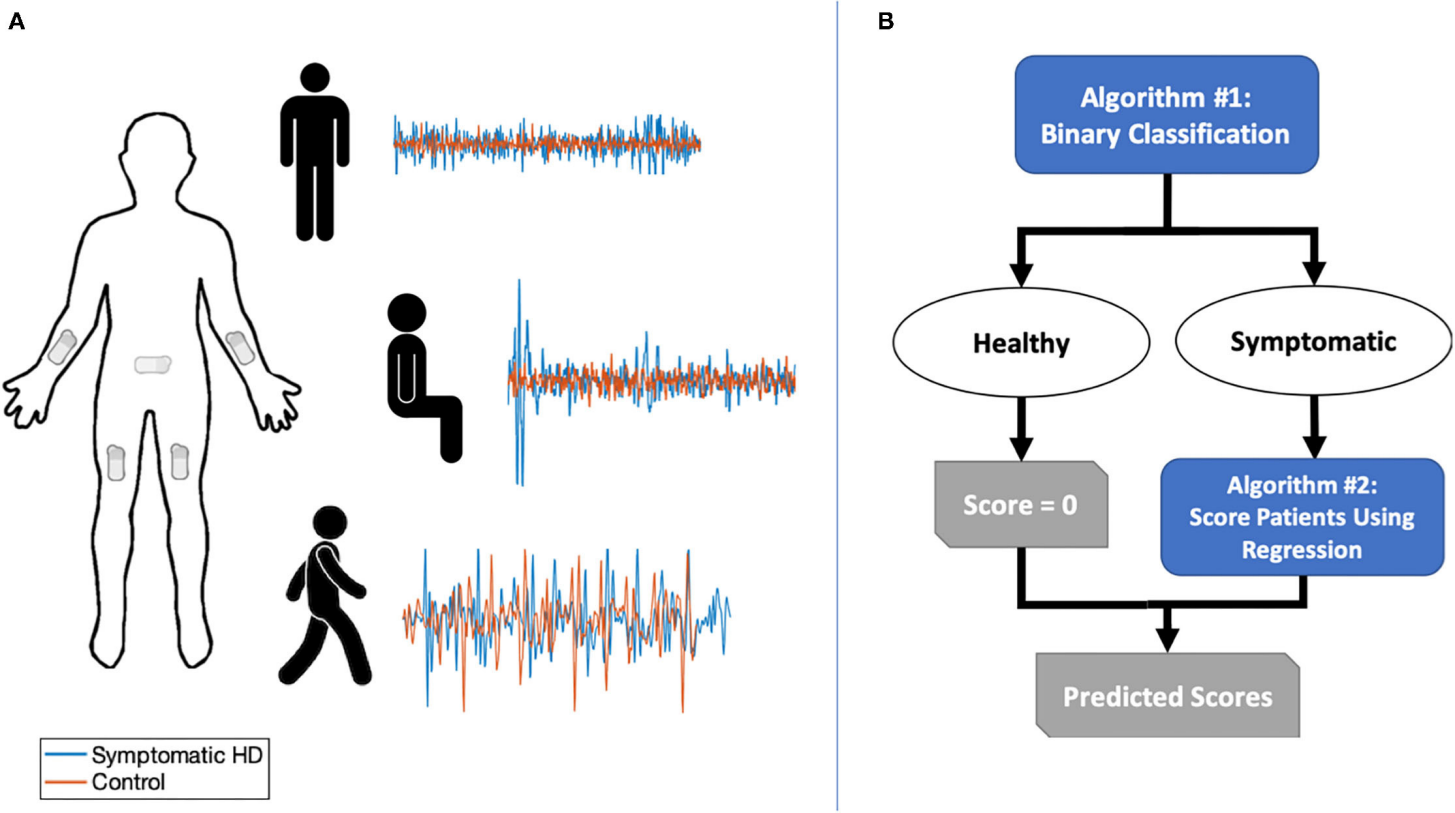
**(A)** Sensor placement and sample of combined triaxial acceleration data for symptomatic HD participants and controls in three tasks: standing with feet apart for 30 s; sitting for 30 s with feet planted and back unsupported; and walking along an 11-yard straight path over five repetitions. **(B)** Two-stage classification and prediction flowchart.

We recruited a convenience sample of 14 individuals with clinically manifest HD and confirmatory genetic testing (>36 CAG repeats) and 14 healthy controls (unaffected caregivers). Participants provided informed, written consent and all study activities were approved by the University of Pennsylvania's Institutional Review Board. Five BioStamp nPoint® sensors (MC10, Inc.; Maguire Rd., Lexington, Massachusetts, USA) were attached to each participant's forearms, thighs, and sacrum, respectively (Figure 1). The BioStamp sensor is a 510(k)-approved, reusable, wearable device equipped with a triaxial accelerometer and gyroscope.

We obtained triaxial linear acceleration signals from all five sensors and angular velocity signals from arm and sacral sensors as the participant performed three tasks: (1) sitting for ~30 s with feet planted and back unsupported; (2) standing with feet apart for ~30 s; and (3) walking along an 11-yard straight path over five repetitions (Figure 1). A neurologist specializing in movement disorders (PGA) subsequently completed the UHDRS-TMS assessment for patients with HD. Healthy controls were assigned a total score of zero.

### Feature Extraction

We split the accelerometer and gyroscope data from each sensor into seven task segments, one segment corresponding to each of the sitting and standing tasks, and five segments corresponding to each straightaway walking interval, ensuring that turns were excluded. The accelerometer and gyroscope data in each segment was pre-processed by first applying a 5th order bandpass filter between 1 and 16 Hz, a frequency range that should capture relevant motion while attenuating signal noise (22). Next we combined the three axial signals for a given sensor and signal type to obtain an orientation-invariant signal as follows:


$$\begin{array}{l} |S_{Total}|=\sqrt{{S_{x}}^{2}+{S_{y}}^{2}+\;{S_{z}}^{2}}, \end{array}$$

where S_(.)_ represents an axial component of the recorded signal. After preprocessing, we were left with eight orientation-invariant signals from the five accelerometers and three gyroscopes per task.

Thirteen summary statistics were computed over the eight inertial segments for a total of 104 features per task. These features were selected based on literature review and clinical judgment of the types of quantitative measures that would capture the abnormal movements observed by clinical HD experts. For each segment, we first calculated the root mean squared, absolute maximum, and standard deviation (SD) of the signal in the segment. Next, we located all peaks in the segment using MATLAB's findpeaks function, and calculated the mean, SD, maximum, and minimum peak height. We also calculated the mean and SD of the inter-peak-interval. Finally, we calculated the average power in the broadband signal, and average band power in three evenly-divided bands of the 6 Hz window surrounding the mean normalized frequency of the broadband signal (23). For example, we would calculate average bandpower in the windows of 7–8, 9–10, and 11–12 Hz for a mean normalized frequency of 9.5 Hz. We averaged the calculated features across segments of the five walking intervals, and thus had 312 features total from the three tasks per patient.

### Predictive Scoring Method

**Figure 2:**
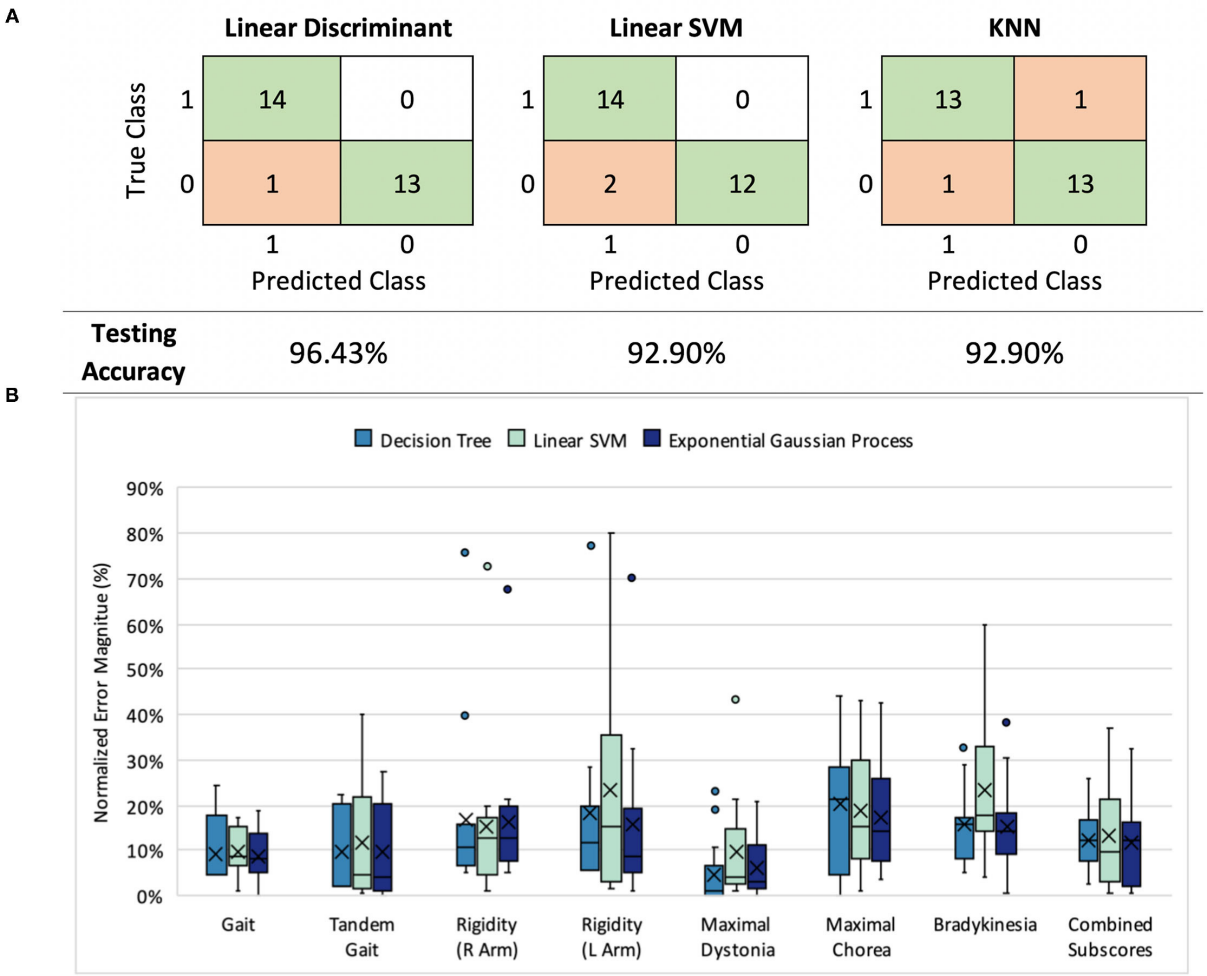
**(A)** Classification results: model performance comparison (healthy controls= 0, symptomatic HD participants=1) **(B)** UHDRS subscore prediction: normalized mean absolute error (nMAE) in predicted score for decision tree, linear SVM, and Gaussian process models in 7 UHDRS subscores and composite subscore. Boxplots indicate the 75% interquartile interval (box), median (solid line), mean (x), maximum and minimum values excluding outliers (whiskers), and individual errors (dots).

We developed a two-stage machine learning method to predict UHDRS-TMS subscores. The goal of the first stage was to use a binary classifier to discriminate between healthy controls and participants with HD. In the second stage, we aimed to predict UHDRS-TMS subscores in participants classified as having symptomatic HD (Figure 2A). The UHDRS-TMS consists of multiple subscores that are rated on a 0–4 scale, including vertical and horizontal ocular pursuit, saccade initiation, saccade velocity, and tongue protrusion. Because our sensors would not directly record any eye or face motion, we chose to predict seven specific UHDRS-TMS subscores related to our sensor placement—left and right arm rigidity, total chorea, total dystonia, bradykinesia, gait, tandem gait— as well as a composite sum of the seven subscores.

For the first stage of our model we trained and evaluated three types of binary classifiers–a linear support vector machine (SVM), a linear discriminant analysis (LDA) model, and a K-nearest neighbors (KNN) model–to compare different classification mechanisms. We used leave-one-out (LOO) cross validation to train and validate the three models; across 24 training rounds we held out one participant as a test set and used the remaining participants for model training. In each round, we first performed feature selection using the least absolute shrinkage and selection operator (LASSO) to reduce the number of features from 312 to fewer than 20 features (24). Finally, we used the trained model to predict the class (HD symptomatic or control) of the held-out participant. The MATLAB functions fitcsvm, fitdiscr, and fitcknn were used for model training.

For the second stage, we trained linear SVM, decision tree, and exponential gaussian process models with training data restricted to symptomatic HD participants. We separately predicted the seven selected TMS-UHDRS motor subscores (left and right arm rigidity, total chorea, total dystonia, bradykinesia, gait, and tandem gait) and a composite sum of the seven subscores. Again we used LOO cross-validation to train and validate the models across 14 training rounds. LASSO was used to first select relevant features in each round, and then the TMS-UHDRS subscore was predicted for the held-out participant. The MATLAB functions fitrsvm, fitrtree, and fitrgp were used for model training.

Finally, we chose the best performing model from each stage together to complete our full model. We performed LOO cross validation to obtain a TMS-UHDRS prediction for each participant. Participants classified as controls in the first stage were assigned a score of zero, and the TMS-UHDRS subscore was predicted for the participants classified as HD patients.

### Model Performance and Statistical Analysis

We evaluated the performance of the three binary classifiers by comparing the predicted class of the held-out participants to their actual class assignment; we calculated accuracy (percent correctly classified), sensitivity (true positives/total positives), and specificity (true negatives/total negatives) of each classification model.

To assess the performance of the second stage of our model, we computed the absolute difference between the predicted subscore and true subscore for each held-out patient. Then we normalized that value by the range of the subscore to account for different subscore ranges (e.g., chorea has a range of 20: four possible points for each arm, each leg, and the trunk), and averaged the error across patients. We termed this value the normalized mean absolute error (nMAE), with equation given as


$$\begin{array}{l} nMAE\;=\;\frac{1}{N^{*}r}\;\overset{N}{\underset{i=1}{\sum }}|y\left(i\right)-\;ŷ\left(i\right)|, \end{array}$$

where *N* is the number of patients, *y(i)* and ŷ(*i*) are the predicted and true scores for patient *i*, and *r* is the score range. We also used the nMAE to evaluate the performance of the full two-stage model.

The non-parametric Mann-Whitney *U*-test was used to determine whether the absolute error in subscore predictions was significantly different between pairs of prediction models. Pearson's correlation was used to determine whether model error corresponded to external patient factors.

## Results

### Stage 1—Classification Performance

Three first-stage classification models (LDA, linear SVM, and KNN) were compared by their ability to classify HD symptomatic participants and control participants (Figure 2A). Each model exhibited a LOO cross-validation accuracy of at least 92.9%, with our LDA model demonstrating the best performance (96.4% accuracy, 92.9% sensitivity, and 100% specificity). The same 11 features were included in at least 50% of the feature sets selected during cross-validation. The selected features were not categorized by a particular task, signal type, or sensor location, but instead represented all tasks, signals, and most sensors (Supplementary Figure 2).

### Stage 2—Scoring Performance

Next, we assessed the performance of decision tree, linear SVM, and exponential Gaussian process models in predicting each of the seven UHDRS-TMS subscores and the composite subscore. We were curious about whether our data and features would perform better for some subscores over others. We also wondered whether the most accurate way to predict the composite subscore was to predict each subscore separately and add them together, or to directly predict the full composite subscore value.

We compared all three models across subscore predictions using the nMAE reported as a percent of score range (Figure 2B), and found that all three performed similarly (Mann-Whitney *U, p* > 0.05). In both decision tree and Gaussian process models, the nMAE for gait subscores and dystonia fell under 10% of the possible score range. Bradykinesia, chorea, and arm rigidity were more difficult to predict individually, with the nMAE ranging from 17 to 25%. We also found that combining predicted scores from the seven subgroups to predict the composite UHDRS subscore resulted in a higher nMAE (Gaussian process, absolute error Mdn = 27.4%, Q1 = 23.9%, Q3 = 32.5%) than did training a single model to predict the composite subscore (Gaussian process, absolute error Mdn = 12.3%, Q1 = 3.0%, Q3 = 14.9%).

### Total Model Performance

**Figure 3:**
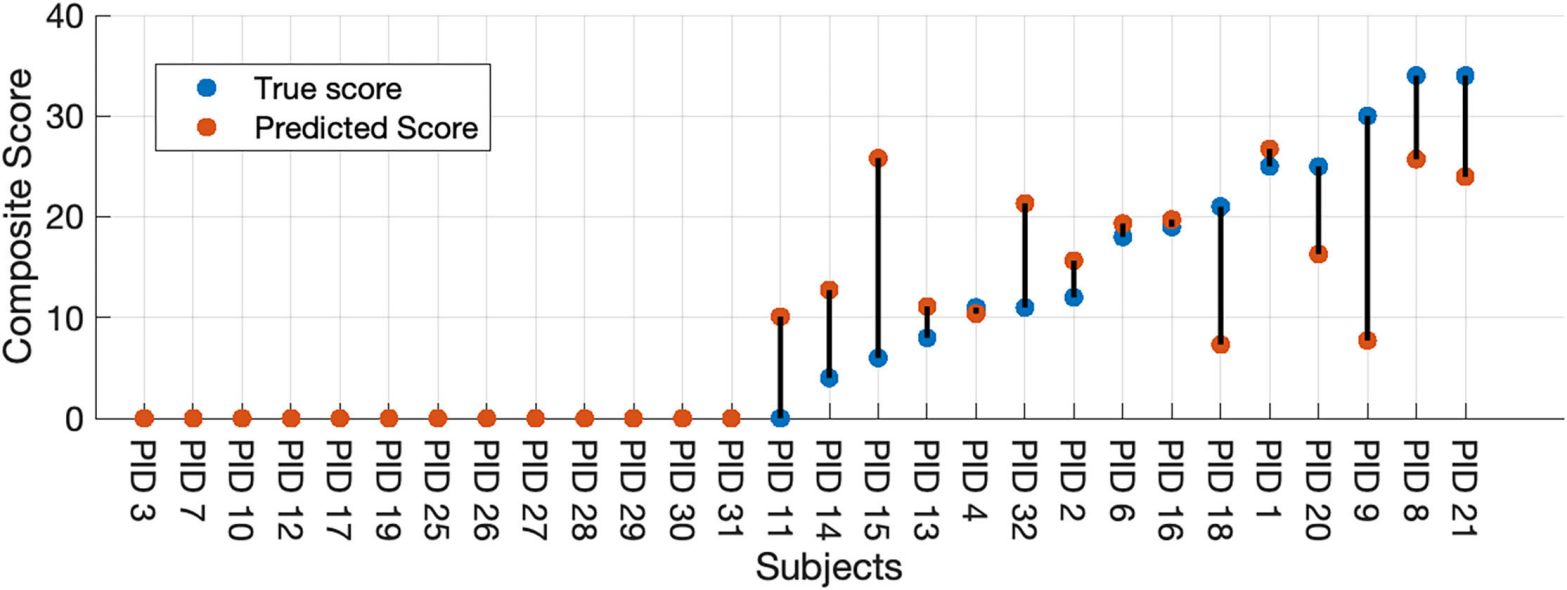
Full-model composite score prediction. Full-model prediction of the composite UHDRS-TMS subscore vs. true subscore for all participants. The composite UHDRS-TMS subscore is a sum of the of the left and right arm rigidity, total chorea, total dystonia, bradykinesia, gait, and tandem gait subscores.

Finally, we used our two-stage machine learning method to (1) discriminate between healthy controls and participants with HD, then to (2) predict the seven selected UHDRS motor subscores and composite subscore in participants classified as having symptomatic HD. We combined results from the LDA binary classifier and selected the Gaussian process model to measure the full-model performance. All thirteen participants classified as healthy by the LDA model received a score of zero. A UHDRS-TMS subscore was predicted for the single misclassified control participant, and the predicted score was included in the error analysis (Figure 3). The nMAE for predicting the composite UHDS subscore using the full model was 6.37% (Mdn = 0.97%, Q1 = 0%, Q3 = 12.64%) of the score range. We found no significant association of total model prediction error and age, total UHDRS-TMS score, or UHDRS composite subscore (Pearson's correlation *p* > 0.05).

## Discussion

We present a quantitative machine learning method that uses biometric sensor data to identify participants with HD and to predict UHDRS-TMS subscores. Our work complements prior studies that have used longitudinal data, more tasks, or greater task complexity to objectively quantify motor features in HD (4, 17, 18). We demonstrate that with <5 min of data over three simple tasks, we can discriminate between participants with symptomatic HD and healthy controls with an accuracy that is comparable to previous work that used longer recording durations or tasks with greater complexity (4, 17, 23).

Although decision trees and linear SVM models have demonstrated predictive power in other studies, we found that LDA and Gaussian process models performed as well or better with our particular feature set (17, 18). Signal features extracted from both the linear accelerometer and gyroscope were selected in all optimized feature sets, suggesting that angular velocity may be a promising addition to previously reported models that rely on linear acceleration alone. Our prediction error was smallest for subscores encompassing dynamic, high-frequency movements evident during gait and chorea assessments. The relatively high prediction error for subtle or slow changes (i.e., bradykinesia), is likely due to a combination of sensor placement and task brevity.

Outliers were observed in the prediction of dystonia and arm rigidity for some patients. The outliers may be due in part to intrarater variability during visual scoring of dystonia and arm rigidity or due to variability in sensor placement along the limbs (3). Bradykinesia, chorea, and arm rigidity may be more easily predicted in future studies by adding more sensors (e.g., shins, thighs, forearm, and triceps). Additional training data representing a broad range of patient severity levels would likely improve the prediction accuracy.

### Limitations

This study has several limitations. First, only participants with HD who were independently ambulatory were included, which may limit the generalizability of our predictive models to patients with more advanced disease. Second, the small size of our dataset may result in high variance between predictive models and the possibility of overfitting a model to our sample data, also resulting in poor generalizability of our selected model. To mitigate the possibility of overfitting, we evaluated model performance using LOO cross-validation, which should yield an approximately unbiased estimation of model performance for future data (25).

The wireless nPoint bioStamp sensors used in our methods were easy to implement in a clinical setting, and eventually the devices could be used to monitor the severity of abnormal movements throughout daily living or while sleeping. Currently there are certain UHDRS categories that our algorithms would not accurately predict (ocular pursuit, saccade, pronation, luria, etc.) with the current sensor configuration. Sub-categories might be quantified in future experiments by adding sensors to hands, feet, and neck.

One of the objectives of this study was to perform classification of patients using only small amounts of data, easily obtained in a routine office visit with little provider time required. As a result, classifications were performed on a comparatively small dataset. It is anticipated that adding more longitudinal data, such as from at-home recordings will lead to more accurate classification results. Despite the short data sampling periods and small numbers of patients, classification results compared favorably to reported interrater reliability in UHDRS-TMS measurements.

### Future Work

The methods outlined in our study may be suitable to validate digital candidate biomarkers used in clinical trials and in clinical decision-making. Ultimately, a complement of sensor configurations and analysis algorithms combining both longer term measurement periods and briefer point measurements of motor features, as employed in this study, may provide for richer objective motor phenotyping of HD. Future directions include evaluating these methods with additional HD patient populations including prodromal and presymptomatic HD, juvenile-onset HD, and late-onset HD. We also plan to capture longitudinal data from patients in the home and community settings. We share all our data in a cloud repository for collaboration to accelerate these efforts.

## Conclusion

Our prediction protocol requires minimal training to implement and could benefit future studies which seek to monitor HD-severity, progression, or therapeutic response in a non-clinical setting. The results of our study demonstrate the feasibility of applying machine learning methods to movement data collected during brief, simple tasks in the classification of abnormal movement in HD.

## Data Availability Statement

The raw data supporting the conclusions of this article will be made available by the authors, without undue reservation.

## Ethics Statement

The studies involving human participants were reviewed and approved by the Hospital of the University of Pennsylvania's Institutional Review Board. The patients/participants provided their written informed consent to participate in this study.

## Author Contributions

SA and PG-A conceived the study. SA, RP, and PG-A collected the data. BS, SA, RP, IT, and SB designed and executed the computational analysis. All authors were involved in preparing the manuscript for submission.

## Funding

This work was supported by the Mirowski Family Foundation, Jonathan Rothberg, Neil and Barbara Smit, and NIH 4UH3 NS095495.

## Conflict of Interest

PG-A is currently employed full-time at Spark Therapeutics, however this work was completed while he was a full-time faculty member at the University of Pennsylvania, has a consulting agreement with SAGE Therapeutics, has licensed intellectual property to Spark Therapeutics *via* the University of Iowa and received consulting fees from Acorda Therapeutics, all unrelated to this work. The remaining authors declare that the research was conducted in the absence of any commercial or financial relationships that could be construed as a potential conflict of interest.

## Publisher's Note

All claims expressed in this article are solely those of the authors and do not necessarily represent those of their affiliated organizations, or those of the publisher, the editors and the reviewers. Any product that may be evaluated in this article, or claim that may be made by its manufacturer, is not guaranteed or endorsed by the publisher.

## References

[1] Huntington Study Group. Unified Huntington's disease rating scale: reliability and consistency. Mov Disord. (1996) 11:136–42. 10.1002/mds.870110204 8684382

[2] Mestre TA Forjaz MJ Mahlknecht P Cardoso F Ferreira JJ Reilmann R. Rating scales for motor symptoms and signs in Huntington's disease: critique and recommendations. Mov Disord Clin Pract. (2018) 5:111–7. 10.1002/mdc3.12571 30363393 PMC6174417

[3] Winder JY Roos RAC Burgunder J-M Marinus J Reilmann R. Interrater reliability of the unified Huntington's disease rating scale-total motor score certification: interrater reliability of the UHDRS-TMS. Mov Disord Clin Pract. (2018) 5:290–5. 10.1002/mdc3.12618 30363437 PMC6174470

[4] Dinesh K Snyder CW Xiong M Tarolli CG Sharma S Dorsey ER. A longitudinal wearable sensor study in Huntington's disease. J Huntingt Dis. (2019) 2019:1–13. 10.3233/JHD-190375 31868675

[5] Farzanehfar P Woodrow H Braybrook M McGregor S Evans A Nicklason F. Objective measurement in routine care of people with Parkinson's disease improves outcomes. Npj Park Dis. (2018) 4:46. 10.1038/s41531-018-0046-4 29644334 PMC5882961

[6] Heldman DA Harris DA Felong T Andrzejewski KL Dorsey ER Giuffrida JP. Telehealth management of Parkinson's disease using wearable sensors: an exploratory study. Digit Biomark. (2017) 1:43–51. 10.1159/000475801 29725667 PMC5927622

[7] Kegelmeyer DA Kostyk SK Fritz NE Fiumedora MM Chaudhari A Palettas M. Quantitative biomechanical assessment of trunk control in Huntington's disease reveals more impairment in static than dynamic tasks. J Neurol Sci. (2017) 376:29–34. 10.1016/j.jns.2017.02.054 28431622

[8] Ye Q Xia Y Yao Z. Classification of gait patterns in patients with neurodegenerative disease using adaptive neuro-fuzzy inference system. Comput Math Methods Med. (2018) 2018:9831252. 10.1155/2018/9831252 30363986 PMC6186329

[9] Baratin E Sugavaneswaran L Umapathy K Ioana C Krishnan S. Wavelet-based characterization of gait signal for neurological abnormalities. Gait Posture. (2015) 41:634–9. 10.1016/j.gaitpost.2015.01.012 25661004

[10] Zhan A Mohan S Tarolli C Schneider RB Adams JL Sharma S. Using smartphones and machine learning to quantify Parkinson disease severity the mobile Parkinson disease score. J Am Med Assoc Neurol. (2018) 75:876–80. 10.1001/jamaneurol.2018.0809 29582075 PMC5885192

[11] Reilmann R Bohlen S Kirsten F Ringelstein EB Lange HW. Assessment of involuntary choreatic movements in Huntington's disease-toward objective and quantitative measures. Mov Disord. (2011) 26:2267–73. 10.1002/mds.23816 21661053

[12] Sen-Gupta E Wright DE Caccese JW. Wright Jr JA, Jortberg E, Bhatkar V, et al. A pivotal study to validate the performance of a novel wearable sensor and system for biometric monitoring in clinical and remote environments. Digit Biomark. (2019) 3:1–13. 10.1159/000493642 32095764 PMC7015390

[13] Moon Y McGinnis RS Seagers K Motl RW Sheth N Wright JA. Monitoring gait in multiple sclerosis with novel wearable motion sensors. PLoS ONE. (2017) 12:e171346. 10.1371/journal.pone.0171346 28178288 PMC5298289

[14] Toosizadeh N Mohler J Lei H Parvaneh S Sherman S Najafi B. Motor performance assessment in Parkinson's disease: association between objective in-clinic, objective in-home, and subjective/semi-objective measures. PLoS ONE. (2015) 10:e124763. 10.1371/journal.pone.0124763 25909898 PMC4409065

[15] Maetzler W Domingos J Srulijes K Ferreira JJ Bloem BR. Quantitative wearable sensors for objective assessment of Parkinson's disease. Mov Disord. (2013) 28:1628–37. 10.1002/mds.25628 24030855

[16] Andrzejewski KL Dowling AV Stamler D Felong TJ Harris DA Wong C. Wearable Sensors in Huntington Disease: A Pilot Study. Journal of Huntington's Disease. (2016) 5:199–206. 10.3233/JHD-160197 27341134

[17] Bennasar M Hicks YA Clinch SP Jones P Holt C Rosser A. Automated assessment of movement impairment in Huntington's disease. IEEE Trans Neural Syst Rehabil Eng. (2018) 26:2062–9. 10.1109/TNSRE.2018.2868170 30334742 PMC6196596

[18] Gordon MFF Grachev IDD Mazeh I Dolan Y Reilmann R Loupe PSS. Quantification of motor function in Huntington disease patients using wearable sensor devices. Digit Biomark. (2019) 19355:103–15. 10.1159/000502136 32095771 PMC7011722

[19] Waddell EM Dinesh K Spear KL Elson MJ Wagner E Curtis MJ. GEORGE®: a pilot study of a smartphone application for Huntington's disease. J Huntingt Dis. (2021) 10:293–301. 10.3233/JHD-200452 33814455

[20] van Vugt JPP Siesling S Piet KKE Zwinderman AH Middelkoop HAM van Hilten JJ. Quantitative assessment of daytime motor activity provides a responsive measure of functional decline in patients with Huntington's disease. Mov Disord. (2001) 16:481–8. 10.1002/mds.1097 11391742

[21] Bennasar M Hicks Y Clinch S Jones P Rosser A Busse M. Huntington's disease assessment using tri axis accelerometers. Procedia Comput Sci. (2016) 96:1193–201. 10.1016/j.procs.2016.08.163

[22] Kolarijani MAS Amirsalari S Haidari MR. Analysis of variations of correlation dimension and nonlinear interdependence for the prediction of pediatric myoclonic seizures – a preliminary study. Epilepsy Res. (2017) 135:102–14. 10.1016/j.eplepsyres.2017.06.011 28654830

[23] Jeon H Lee W Park H Lee HJ Kim SK Kim HB. Automatic classification of tremor severity in Parkinson's disease using a wearable device. Sensors. (2017) 17:2067. 10.3390/s17092067 28891942 PMC5621347

[24] Tibshirani R. Regression shrinkage and selection *via* the lasso. J R Stat Soc Ser B Methodol. (1996) 58:267–88. 10.1111/j.2517-6161.1996.tb02080.x

[25] Hastie T Tibshirani R Friedman JH. The Elements of Statistical Learning: Data Mining, Inference, and Prediction. 2nd ed. New York, NY: Springer (2009). p. 745. 10.1007/978-0-387-84858-7

