# Supplementary material for: Predicting Severity of Huntington's Disease With Wearable Sensors
Source: Front Digit Health. 2022 Apr 4;4:874208. doi: 10.3389/fdgth.2022.874208 (PMC9013843; doi:10.3389/fdgth.2022.874208)
Supplement: Supplementary file 1 [file Data_Sheet_1.PDF]

Brittany H Scheid, Meng., Stephen Aradi, MD, Robert M Pierson, MSE, Steven Baldassano, PhD, Inbar Tivon, MS., Brian Litt, MD, Pedro Gonzalez-Alegre, MD/PhD

Supplementary Material for "Predicting Severity of Huntington's Disease With Wearable Sensors"

1 Supplementary Figures and Tables

1.1 Supplementary Figures

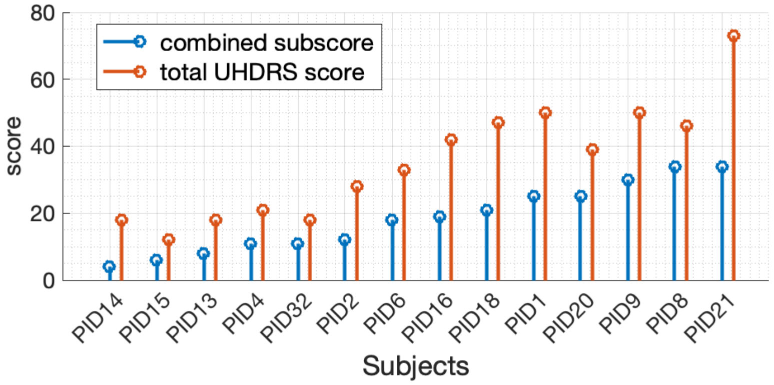

Supplementary Figure 1. UHDRS-TMS score distribution across patients in this study. The total UHDRS score is out of 124 points, and the combined subscore is out of 68 points and includes the bradykinesia, rigidity (L/R arm), gait, tandem gait, dystonia, and chorea subscores for all four limbs.

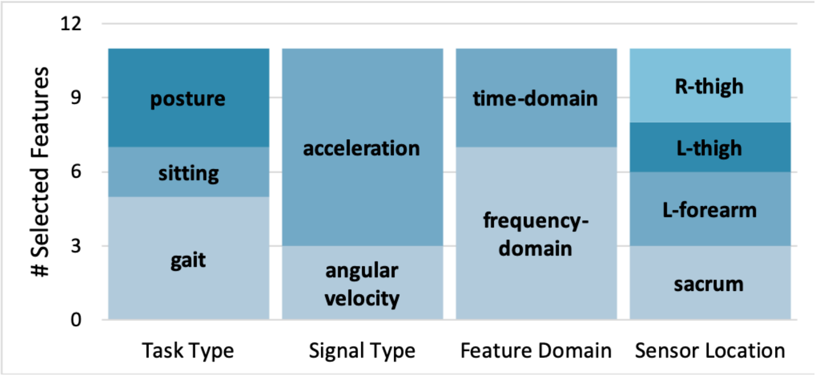

Supplementary Figure 2. Selected Feature Characteristics. Attributes of the 11 features included in over 50% of the feature sets selected in leave-one-out cross-validation. Data from all tasks and both sensor modalities were present after a LASSO feature selection.

1.2 Supplementary Tables

Supplementary Table 1. Patient Information.

	HD	Controls
Total number	14	14
Age (years)	54.6 +/- 10.8	42.0 +/- 19.2
Female [%]	64.3	21.4
White [%]	91	100
African American [%]	9	0
% taking anti chorea medication	81.80%	0.00%
% who started or increased anti chorea medication	55.60%	0.00%
Average Total UHDRS-TMS (/124)	35.4 +/- 17.2	0
Average chorea (/28)	9.6 +/- 5.6	0
Average gait (/4)	1.1 +/- 0.7	0
Average tandem (/4)	2.1 +/- 0.5	0
Average dystonia (/20)	2.9 +/- 2.6	0
Average bradykinesia (/4)	1.1 +/- 0.9	0
55 yd time (s)	55.5 +/- 10.2	46.7 +/- 3.6
